# Social Networking of Quasi-Species Consortia drive Virolution via Persistence

**DOI:** 10.3934/microbiol.2021010

**Published:** 2021-04-30

**Authors:** Luis P. Villarreal, Guenther Witzany

**Affiliations:** 1. Center for Virus Research, University of California, Irvine, USA; 2. Telos-Philosophische Praxis, Buermoos, AUSTRIA

**Keywords:** virolution, quasispecies consortia, virus persistence, membership roles

## Abstract

The emergence of cooperative quasi-species consortia (QS-C) thinking from the more accepted quasispecies equations of Manfred Eigen, provides a conceptual foundation from which concerted action of RNA agents can now be understood. As group membership becomes a basic criteria for the emergence of living systems, we also start to understand why the history and context of social RNA networks become crucial for survival and function. History and context of social RNA networks also lead to the emergence of a natural genetic code. Indeed, this QS-C thinking can also provide us with a transition point between the chemical world of RNA replicators and the living world of RNA agents that actively differentiate self from non-self and generate group identity with membership roles. Importantly the social force of a consortia to solve complex, multilevel problems also depend on using opposing and minority functions. The consortial action of social networks of RNA stem-loops subsequently lead to the evolution of cellular organisms representing a tree of life.

## 1. Introduction

A crucial question in the evolutionary tree of life concepts remains how variations, especially genetic variations occur that are object of biological selection. The neo-darwinistic narrative of the last century always assumed that the variation process is the result of random replication errors (mutations). It remained a curious explanation to reconstruct the emergence of organismic complexity by selection of accumulated beneficial errors. In contrast to this we assume both the evolutionary relevant genetic variations are the result of natural genome editing by viruses and related genetic parasites and the transfer and integration of virus derived genetic content into cell based organisms by infection events. Such events were coopted and exapted by the host and integrated remaining viral defectives such as mobile genetic elements and non-coding RNAs served to reprogramm gene expression and epigenetic markings. This we term virolution, virus driven evolution. The application of virolution overall to issues regarding the origin and evolution of life can provide us with a very distinct and new perspective on how living organisms emerge.

## 2. Virus-like genetic parasites can build cooperative networks

The involvement of a virus-like process in the origin of life seems illogical. How can a molecular genetic parasite of the host system (virus) be involved in the origin of the host, even if the host is a more simplified RNA based living system? Would this not require the pre-existence of the host? But the term ‘virus-like’ includes parasites of RNA (stem-loop) replicators, even those simple RNAs that must have been ancestral to any RNA based life forms [1]. Due to high rates of generating diversity, parasitic replicators emerge at the very origins of RNA quasi species [2],[3]. However, parasites have previously been treated as presenting a major problem for origin of life scenarios [4],[5]. Recently, we have come to realize that virus evolution can often involve complex cooperative as well as antagonistic group behaviors [6],[7]. By embracing a fundamental role for diversity and parasites in the origin of life, we can understand how cooperation of both kinds of RNA replicators-very similar ones and even distinct ones-can emerge with more complex function and why this fundamentally generates networks (RNA societies), not fittest type individuals [8]–[10].

## 3. Virolution drives complex adaptations

Virolution means virus as mediators of evolution and as competent agents to edit host code [11],[12]. Virolution in an RNA virus employs consortial, quasispecies swarms that retain swarm (network) identity [13]. By then following the ‘virolution’ (cooperative consortia) of such stem-loop RNA communities, we can better understand the origin of virus, tRNA and the ribosomes [14]–[17]. Virolution also allows us to understand more complex adaptations that affect group identity, immunity and multiple identities such as various cell types [18]. Such complex adaptations can create and modify existing networks [19]. The social force of networks of stem-loop RNAs to solve complex problems did not terminate with the emergence of RNA based life [20].

## 4. Virus mediated evolution: virolution

A ‘virus first’ perspective for understanding human evolution will likely seem counter intuitive or even preposterous to many readers [21],[22]. Surely these most selfish and destructive of agents cannot be proposed to have contributed substantially to the many complex features that make us human. Viruses are genetic parasites, often capable of transmission and dependent on their host for replication and/or maintenance [23]. They are thus fundamentally able to both, interact with and contribute to host genetic and epigenetic content [24]. Both capacities allow viruses to be editors of host genetic content [25]–[27]. We know viruses to be agents of disease, often serious and even fatal. In what way can these capacities relate to the complexity needed to generate capabilities of organisms in all domains of life [28]? Besides the direct interaction on host genomes viruses are also capable of colonizing and persisting in host genomes and becoming one with them with more far reaching consequences [29],[30]. In this case viruses may introduce instruction sets to host cells [31]. Such new and diffuse instruction sets can promote new regulatory networks with new capacities [32],[33].

This process has been called ‘virolution’; virus mediated evolution [34]. And it is the persisting viruses that are highly host specific which, usually sexually transmitted also have the ability to differentially affect host survival. The relationship of persisting viruses to its host population has been proposed to contribute significantly to host survival and affect the whole tree of life [35]–[37]. Such a process is fundamentally symbiogenic [38].

## 5. Consortial, cooperative, multifunctional and transmissive RNA

Why would viruses promote novelty via the formation of complex networks able to contribute to host phenotypes [39]? The currently accepted view is that viruses are simply providing an extended source of errors (diversity) that can occasionally become ‘exapted’ by their host for host purposes [40]. An infected individual host variant will survive and somehow adapt virus information for its own survival [18]. Networks are then created from this information in step wise series of selection events. The real answer, however, lies much deeper then is likely to be appreciated. Indeed, it relates directly to the earliest events in the evolution of life reaching all the way back into the RNA world [41],[42]. This world is characterized by consortial, cooperative, multifunctional and transmissive RNA agents that operate in groups that can identify network membership and preclude non-members which clearly are the forerunners of cell-based immune systems [8]. More recently it was detected that RNAs use signaling molecules which drive RNA communication networks to coordinate cooperative interactions [43].

We have long focused on the modest genetic adaptations associated with neo-Darwinian variation and selection. But whenever a host genome becomes colonized by non-ancestral endogenos retroviruses and related elements that replicate via RNA, a quasi-species consortia mediated process again applies to modify existing RNA networks that provided identity (being necessary for control and immunity) superimposing new and often multiple uses of stem-loop RNAs that are now engaged in and generate new identity networks [18],[44]. This means such a colonizing event may activate posttranscriptional active RNAs out of former conserved functions into a new context of genomic plasticity. This is a much more creative and punctuated process, able to promote complex regulatory shifts, but one that still essentially uses invasive stem-loop RNA agents.

## 6. Definition problems

The term virus has a broad and almost instinctive meaning to many people with respect to disease. It is however, worthwhile exploring a current definition of this term in order to employ it with greater consistency and precision [45]–[47]. Since it is well known that many viruses can infect and exist within their host with no disease, clearly disease cannot be a defining characteristic [48]. Nor is uncontrolled replication a defining characteristic since many viruses have highly regulated replication cycles. Some do encode proteins involved in membrane synthesis. Some do not even encode their own capsid or membrane proteins so this too cannot be a defining characteristic. But so far, no virus has been observed to code for a complete ribosome [17]. Nor do they appear to encode many of the most fundamental metabolic proteins. Thus viruses are fundamentally molecular entities that are parasitic to living systems with ribosomes and energy production. But some viruses are parasitic to other viruses with all their persistent viruses and defectives (thus parasitic to living systems plus virus) and most viruses can generate defective versions of themselves which are parasitic to the host system plus self full virus which represents a never ending modularity [29],[49],[50]. These situations can be very important for some specific viral life styles. Thus our definition must be inclusive of all of these situations. We therefore propose the following characteristics for defining virus:

- A virus is a molecular genetic parasite.
- A virus must be competent in the instruction system of its host system
- A virus must superimpose (edit) new instructions onto the host system (extending the code, bringing novelty, promoting symbiosis)
- Viral instructions must promote maintenance of the virus (i.e. self identity compatible) which includes directed replication needed for either maintenance and/or transmission.
- Virus instructions can also simply include compelling the host to ‘maintain’ the viral instruction set (persistence) and replicate it along with the host.
- Viral instructions must oppose (i.e. damage) competitive instruction sets (i.e., host immunity and/or virus competition).
- These viral instructions may subvert (colonize) and manipulate opposing or competing instruction sets so as to maintain a coherent viral instruction system.
- The simplicity of RNA virus instructions requires that they be a coherent consortia of diverse RNA instructions (QS-C).

In addition to these defining characteristics present in quasi-species consortia we would propose that the original ‘viral’ instruction system were simple stem-loop RNA replicators, as proposed for the RNA world [51]. These parasitic replicators were able to transfer and occupy (ligate) their other RNA stem-loops, including their own quasispecies. Such self invasion promotes the emergence of more complex functions such as ribozymes and a consortial ribosome for example [52],[53]. RNA viruses still depend on these stem-loop instruction agents for basic identity and replication. The host (DNA) has become a habitat for these RNA societies [54].

## 7. Social interactions are not a mathematical expertise

There are other important problems involving definitions that should be mentioned. These include the terms networks and systems. The real problem, relates to attempts to mathematically define these terms so that calculation based approaches can be applied to them [39]. For example, a network stems from the concept of a net, with knots (nodes) connected to each other and originally used in fishing techniques. In network theory this was applied to mathematical modelling. Similarly, formal complex systems posits a mathematical foundation for defining systems [55]. However, in the context of diverse but coherent RNA agents (QS-C), it is not possible to mathematically set either the potential interactions or the nature of these interactions for a single RNA stem-loop as it will have conditional, context and history dependent activities (uses) within the population [56],[57]. Although statistical modelling may generate results to find quantitative traits that are based on unequivocal data, contextual and history dependent properties of quasispecies consortia can be defined unequivocally only in rare cases [58].

However, it does compel us to use the terms networks and systems in a less mathematical (but popular) way. The concept of network in particular will be important for our discussions as it will relate directly to vital group identity which will require the specification of network membership characteristics [59]. For an RNA agent, being a member of a network relates directly to its identity markers (often stem-loops).

We will often consider the issue of group identity and group behavior as these are the proposed to provide the foundations of social interactions. Therefore we seek to define a network from the perspective of a consortia of RNA agents and apply the strategies of these diffuse transmissive agents to explain the creation of new networks and redirecting existing ones [39]. However, it will be very difficult to think about and communicate these consortial or social issues. This is not because they are so inherently complex, but more because they are fundamentally interactive social phenomena that resist a formalizable (mathematical) explanation [60]. A social system will have individual agents such as RNA stem-loops that will fundamentally have multiple (often opposing) activities and uses [61]. This is most apparent in the study of viral QS-C presented below. Social behaviors can be best investigated by analysing action motifs resulting in coordinated group behavior and communication (as documented by e.g., the cells of the immune system) [62]. Especially the social force of communicative interactions cannot be quantified by formalizable procedures, because emerging properties of social (not individual) interactions such as cooperative group behavior depending on contextual an historical circumstances can be better analyzed by social science terminology than by mathematical equations [63].

## 8. Addiction modules: how persistence drives complexity

Another main question is the evolution of complexity. Let us remind the fact, that cellular life represent rare islands in a sea of viruses. As a consequence every cell based life is under constant infection pressure from its beginning until death [64],[65]. Genomic habitats of cellular lifes are a rare resource for genome invading genetic parasites such as persistent viruses, their defectives and an abundance of infection derived mobile genetic elements and non-coding RNAs all characterized by repeat sequence formations, such as transposons, retrotransposons, long terminal repeats (LTRs), non-long terminal repeats (non-LTRs), long interspersed nuclear elements (LINEs), short interspersed nuclear elements (SINEs), Alu's, group I introns, group II introns, plasmids, long non-coding RNAs, miRNAs and many others [66]–[70].

Addiction modules represent at least two competing genetic parasite consortia (viral clouds), which try to invade host genomes. The competition targets not solely each other, but host immune system. This may result both the competing genetic parasites and the host immune system, can together install persistence and remain as a counterbalancing module [71]–[73]. The most interesting aspect here is, that if these persistent agents are conserved into the genomic identity of the host, the former identity changes dramatically. New features are part of the host genomes which did not exist before. In such addiction module integration events, up until 100 new genes can be transferred into the host genome in a single event [18]. This means a rather complex genetic set may be transfered into a host genetic identity via a single infection event.

For example, in the case of a restriction/modification module, this means 52 restriction enzymes are counterbalanced by 52 modification enzymes. This indicates how complex addiction modules may be constructed [74],[75]. One of the best known addiction modules in bacterial life is the restriction/modification (R/M) system, which is a common feature with immune functions. One part consists of an antitoxic modification enzyme, which represents an unstable protective agent. The counterpart consists of a toxic restriction enzyme, which is a stable but destructive. [71],[76].

Most importantly, this new information representing genetic novelty by a persistent integration of counterbalanced coded genetic elements is not the result of error replication, but a result of module-like linked genetic contents. This fundamental difference to error replication narratives proposes new nucleic acid sequence constructions by integration of larger content arrangements into a coherent syntax without destroying the already existing sequence content [77]. The explanatory model of the last century in which error prone replication of quasi-species dominated, now can be revisted into quasi species consortia with an inherent never ending capability of creative productivity. ‘Error-treshold’ in this perspective is overproduction that cannot be conserved or serve as beneficial genetic content.

This is an unique social force, because several RNA stem loop consortia cooperate by competition together with the host immune systeme and change host genetic identity by complementing into a counterbalancing module. The new capabilities that derive from integrated addiction modules are absent in the same host species which are not object to such infection events. Both, the competing viral clouds and the host genetic identity reach a cooperative upgrade of genetic complexity and addictive dependency on such infection events. Contextual circumstances (stress, environmental change, etc.) may destabilize such addiction modules and counterbalance may get weak or even out of control, the destructive toxin element may become active again and harm or even kill host.

## 9. Networks of non-related infectious agents constitute information in host genomes

The development of neo-Darwinian thinking in 1930's stems directly from the foundation that natural selection acting on variation (mostly from replication errors) in individuals selects for the survival of fittest type variant. Thus the variation in offspring originates from the direct ancestor to the selected individual [78]. However, when ‘non-ancestor’ virus derived genes are seen to occur in host genomes, it is typically reasoned that such genes simply represent another form of variation (errors) that were also somehow associated with individual survival. The surviving host individual was then able to adapt (exapt) these genes for its own purpose and survival [79].

This explanation still invokes a central role for individuals. What then results are various scenarios, such as kin selection, or arms race ideas involving a serial one-upmanship and linear process of selection. Any networks that emerge will then need to stem form this same serial process. The process is not prone to punctuated bursts in evolution nor is it particularly prone to rapid emergence of complexity or novelty. Also, any associative or group behavior that emerges, such as altruistic behavior, will similarly stem indirectly from individual survival, as described by various kin selection or game theory models. This view has been well accepted for numerous decades and many current evolutionary biologist no longer question its basic tenants. Some even like to think of this as laws of evolution. But this is a view that emerged well before we understood the broad and ancient prevalence of virus [27]. In the last several decades analysis of comparative genomics and metagenomic sequencing of numerous habitats has shown us that virus derived sequences dominate in all habitats so far evaluated [80].

The term virosphere has been introduced to describe this vast cloud of genetic information [81],[82]. And within the genomes of organisms, virus derived information is almost always the most dynamic component of host DNA for all domains of life [83],[84]. Much of this virus derived host DNA has long been seen to play no useful role, it was ‘junk’ DNA that was the product of selfish replicators [85],[86]. Yet much of this ‘junk’ was clearly viral derived and often its expression was associated with reproductive tissue. More recently, such ‘junk’ has seemed much more important for the functioning of the organism [87],[88]. But it is still basically seen as ‘exapted’ stuff, put to some inadvertent good host use following individual selection. But the existence of a vast virosphere should compel us to think differently about virus derived information in host genomes. All domains of life must survive in this ancient, unrelenting and extremely adaptable virosphere.

## 10. Cooperative RNAs

How does virolution affect host evolution? Can it be providing some core, essential function needed for life? And more fundamentally, does virolution operate with additional principles, such as consortial group identities (QS-C) that can colonize and transform host, which fundamentally promote networks and complexity? If so, can these principles help us better understand the origin of life.

Such virolution is what promotes the creation of new ‘systems’, not serial selection from errors [89],[90]. But this looks like errors since most of the instructions are subviral [91]. Viruses, the ultimate and nearly invisible selfish agents have finally taught us about the social force of consortia. It is a big lesson and it applies to all levels and eras of life. But why would a consortia of viral agents act to promote complexity? It is for the sake of superimposing group identity and group survival?

The QS-C has to incorporate a new viral derived identity onto the host [92]. This colonization will also clearly affect host survival in its extant virosphere via updated immune functions [93]. The virosphere matters for the success of all life. Such a colonizing event must promote the survival of this information and new viral identity/ecology that results. This is a very different perspective then that of selfish individual type selection. And although virolution supports various forms of multilevel selection, it does not conflict with traditional individual type selection which emerged with DNA based cells and virus. But whenever infectious sets of RNA based replicating agents successfully colonize a host, they will again bring to bare the creative, cooperative and distributed power of QS-C selection to their host [94]. This is a most ancient process that still operates on DNA, using DNA as a stable habitat. The RNAs have multiple regulatory capacity which leads to a better understanding RNA cascades and networks, which are the products of or promoted by serial colonization of virus (and often provide antiviral activity). These regulatory stem-loop RNAs will mostly occupy introns, 3′ UTR and some 5′ promoter regions. We will aso see that older identity/regulatory systems become subjected to manipulation (repurposed) or elimination following successful colonization.

## 11. Fittest individual type reconsidered

RNA viruses have long been recognized as distinct agents from their host cells in that they were the sole survivors of the RNA world that still used RNA as a genetic molecule [95]. That they could replicate so readily and be characterized in the laboratory made them ideal systems to study variation in RNA replication [96]. The variation was considered to result mostly from copy errors of a low fidelity polymerase. And since viruses could be ‘cloned’ they apparently adhered to the concept of individual fittest type selection. Since it was realized early on that RNA replications at the dawn of life in the RNA would also replicate with high error rates, this seemed to present a problem for the origin of life and the origin of the genetic code [97].

It was from this perspective in the 1970's that Manfred Eigen developed the quasispecies equations to explain the quantitative behavior of RNA populations that were generated via errors of the master fittest individual type template [79]. The basic assumptions were then that there was a master fittest type RNA template that would generate a cloud of RNA progeny due to copy errors, but that this cloud would have certain overall behaviors (such as error threshold). Many more theoretical papers followed this early publication by Eigen, by his colleagues representing the exploitation of the formal mathematical analogy of quasispecies dynamics and statistical mechanics [98]. This should finally lead to a theory of evolution based on biochemical kinetics [78]. And in the following decades, a large number of laboratory studies by RNA virologist sought to evaluate and measure various aspects of quasispecies theory [99]. It became very clear that the quasispecies behavior of RNA viruses was very important for understanding clinical outcomes of human infections. And indeed, some of the insights of quasispecies theory were observed, such as error threshold. The concepts of variation of the master fittest type became entrenched during this period as there seemed to be no conflict with more traditional neodarwinian selection.

## 12. The example of retrotransposon activity in human brain evolution

Current knowledge about the evolutionary origin of placenta organ in mammals clearly indicates natural genome editing by persistent retroviruses [100]. Another intrugiung example is human brain evolution. For many years, molecular biologist assumed that the complex RNA expression patterns observed by various techniques (such as hybridization kinetics) in the mammalian brain was due to the expression of many genes, which was expected for such a complex organ [101]. However, comparative genomics has made clear that gene transcription differs little between human and great apes [102]. Indeed total gene numbers differ remarkably little between the simplest animals (C. elegans) and humans. But by far the biggest differences between human and chimpanzee genomes were due to indels (insertion and deletions) [103]–[105]. The great majority of these indels are the result of retrotransposon activity of various types such as ERVs, LTRs, LINES and alus being most numerous [106]. Of these, the alu elements and transcripts are particularly active and affecting RNA editing and intron splicing in the human genome [107]. In addition, they are frequently involved in epigenetic control and can emerge or expand rapidly in genomes [108].

Such a large scale retroposon colonization would seem to pose a highly genotoxic situation for the human genome, an idea which seems supported by genomic analysis [109],[110]. And yet this noncoding DNA is species specific [111], evolving quickly in humans [112], but also appears to be under very strong selective constraints [113],[114]. This seems problematic in several ways: this is an inherently destructive event that should seldom result in novel or complex phenotype, plus it is both rapidly changing between species yet sometimes highly conserved. Indeed, this high rate of change was previously used to argue for the idea that it must be junk DNA. Yet, these are the changes that must be addressed and included to explain the emergence of the large and social human brain [115]. How then can we understand the origin of the most complex organ known (human brain) in the context of such massive introduction of errors? Clearly we cannot. But perhaps the concept of ‘errors’ is itself in error as implied above [116]. Indeed a major correction in our thinking has emerged from the ENCODE project. This project is a consortia of researchers that has sought to characterize all the RNA transcribed from the human genome, including RNA that is not cytoplasmic polyadenylated mRNA but is non-coding RNA [117],[118].

It is now quite clear that most of this ‘junk’ is transcribed and that 95% of the transcripts are from repeated sequences that were retrotransposed [119]. These transcripts include a previously poorly studied class of long non-coding RNA [120],[121]. Furthermore, these non-coding transcripts appear particularly relevant to human brain and cognitive development and evolution [122],[123]. Additionally, long term memory also seems to use non-coding RNA [124]. These observation have led John Mattick to propose that genetic programming in higher organisms (including human) has been misunderstood for 50 years [117]. Regulatory RNA derived from retrotransposons is key to eukaryotic complexity, compelling us to abandon the concept of selfish junk DNA. But in this realization we also come to realize this regulatory RNA is operating mostly as stem-loop RNAs that have complex, multilevel and even opposing functions. It is clearly operating and evolving as a network. But networks of stem-loop RNAs are also thought to have been crucial for the origin of RNA based life [125],[126]. Could it be that the creative social force of networks of stem-loop RNAs involved in the origin of life are still at work during recent human evolution? If we look at the synaptic plasticity in humans, arc-a key protein in memory storage-derived from a retroviral infection event [127],[128].

## 13. To make a network from a collective: quasi-species-consortia (QS-C)

In the ensuing several decades, many laboratory observations were made that indicated more complex collective behaviors for viral quasispecies then were predicted by the quasispecies equations [99]. The most recent compilation of these studies outlines many of the collective behaviors that have been made with quasispecies [40]. The culmination study that most clearly reported that quasispecies have more complex collective behaviors was the study from the Andino group of poliovirus pathogenesis in a mouse model in which diversity and cooperation were key to viral fitness [129],[130]. The quasispecies collectives have distinct and measurable fitness.

- They can compete with and exclude related populations.
- They have minority populations that are crucial for overall fitness [131],[132].
- They can display heterogeneity important for fitness that is not observed in the consensus type [133].
- They can suppress their own replication through lethal defection [134].
- They can be composed of members that can complement and interfere with replication of the collective and many of these features can be observed in clinical infections such as humans with hepatitis C virus [135].

Thus quasi species are collectives that have positive and negative interacting members that are bound together for a combined fitness that depends on diversity [136]–[138]. It is thus ironic in that it is from the viruses, the most selfish of all genetic entities, we experimentally observe the characteristics of cooperative, collective behavior. And it was the ‘fittest type’ assumptions of Eigen that generated quasispecies equations and theory which stimulated the development of this modern collective quasispecies view. But we are left with a conceptual contradiction. Modern quasispecies observations do not depend on the master fittest type and the consensus sequence may not predict to the fitness of the diverse collective. Diversity itself seems crucial.

Such dynamic diversity allows a population of otherwise rather simple agents (such as HIV-1) to defeat a highly complex and evolved system of adaptive and innate immunity in their human host [139]. If such infections were limited to the fittest type individuals, they would fail to overcome such a complex system. Not only can the social force of quasispecies defeat our human immune system, it has also largely defeated our combined human technology by frustrating the development of effective vaccines for 30 years. All this impressive biological competence from a small and ‘simple’ virus. The term QS-C will indicate a ‘collective’ of ‘cooperative’ character to the population. That way the original term, quasispecies (QS), can still apply to fittest type models.

With this clarification, it should become apparent that all RNA replicators (especially simple ones) will have high rates of diversity generation (not error). In contrast to the error replication narrative such high rates of diversity generation can be termed also as high levels of non-directed creative productivity reminding us that living agents in populations does not replicate mechanistically in a machine-like manner but may search also for innovative solutions for unexpected context [89].

In addition, all genetic entities that replicate via RNA will also be prone to QS-C social (collective) behaviors [140]. Importantly these behaviors will include both cooperative and competitive interactions, even within the same individual molecule. RNA, however, is not simply providing a syntax for genetic information. It is more than code. It can also provide structure (stem loop), identity (stem-loops, 5′, 3′ ends) and functional (ribozyme) activity. And it can be dynamic (e.g. pseudoknots) and responsive to the environment (riboswitches) and even frameshifting. A ribosomal frameshift is a natural technique to process alternative translation of an mRNA sequence by changing the open reading frame [141]–[143].

Because of this much extended capacity relative to DNA, RNA can be considered as more active agents, with group behaviors that make it able to function as an agent-based population to affect its own activity and survival [11],[26]. It was from this perspective that we proposed that DNA should be considered as a habitat for these active RNA agents [54]. But this discussion of simple RNA replicators suggest that the concept of QS-C should also apply to the ideas and experiments concerning the ‘RNA world’ hypothesis. Yet curiously, very little ‘RNA world’ research has addressed any issues regarding quasispecies, see [144],[145], let along the more modern QS-C idea. As many are starting to think that life originated in a cooperating situation [146].

## 14. Virolution drives the origin of life

To evaluate the QS-C and infectious perspective on the RNA world hypothesis, we will apply and explore the RNA-agent concept introduced above to the role of stem-loop ribozymes in the origin of life. The main objective is to incorporate the historically absent QS-C and parasitic perspective (with its inherent feature for group fitness) into the process that creates social RNA networks out of prebiotic elements. We will not explore early chemical evolution that might have led to the emergence of RNA molecules but will instead assume RNA has come into existence and follow its features from this perspective. One immediate consequence of this perspective is that we will be focused on collective features of RNA populations and will thus evaluate the chemical consequence of ribozyme QS societies, not individual replicators.

This foundation immediately creates a situation in which collectives of molecules with multiple behaviors will have the primary role in promoting the origin of life. It will also be important early on to consider how these systems maintain coherence (group identity), as this is an essential feature. Indeed, a basic and continuing theme will be that a core function of stem-loop RNAs is to provide molecular identity through all of evolution, including recent human evolution [147].

The idea is then that individual members of stem-loop RNA populations were collectively able to invade (ligate into) each other to form a more stable and capable (ribozyme active) consortia with emergent, transformative and unpredictable abilities. These collective would lead to the origin of various ribosome and other RNA groups within cellular organisms still linked to its stem-loop tRNA origin [148]–[150]. Such a scenario also introduces the basic role of cooperation in the origin of life and thus the communication of RNA stem loops [43]. It does not, however, eliminate competition, preclusion or extinction which are also inherent features of QS-C behaviors. Competition is not disolved, but preliminary counterbalanced by a sophisticated creation of flexible hierarchies. Furthermore, the identity and transmissive role for stem-loop RNAs sets the early (precellular) foundation for the origin of viruses whose emergence will further drive host evolution via persistent colonization. The cooperative and parasitic features of QS-C will also promote the early participation of peptides in the identity and evolution of the abundant groups of ribonucleoproteins [151]. The maintenance of these RNA societies as a coherent collective will generally be mediated by addiction modules, which underlie group identity and immunity in all living systems. With this foundation, the emergence of genes, DNA, cells and individual fittest type selection can all be coherently described although alternative concepts contradicting the RNA-world first hypothesis are still discussed [152]. But the emergence of DNA and cells and Darwinian evolution does not terminate the central role for transmissive RNA societies in the evolution of life. DNA becomes a habitat for these stem-loop ‘identity’ RNAs. One issue should already be clear. This scenario posits that collective and cooperative behaviors were and remain essential for the emergence of living complexity [41].

## 15. How RNA hair-pins generate identity networks

On the origin of the RNA world, short RNA oligomers formed by chemical processes needed to become longer RNAs able to perform template based catalysis. It has been proposed that the initial chemical formation of hairpin-like RNAs (stem loops) could provide ribozyme activity following a ligation based modular evolution that would yield ribozyme auto catalysis [28],[153],[154]. But according to the parameters of QS-C evolution, for a consortium of RNA stem-loop replicators to survive, they must form a coherent population. They must share their identity and survival [19]. The identification of the stem-loop sequence itself by catalytic agents could provide such common identity. Alternatively, chemical markers or initiators of catalysis could also mark the common population for priming or replication. Thus it is very interesting that the smallest ribozyme so far reported consists of just 5 nucleotides able to catalyze aminoacylation of the 3′ end [155],[156]. The addition of an amino acid to an RNA molecule has many interesting chemical implications. A ribozyme has rather limited chemical potential compared to proteins. This is mostly due to proton dissociation constant of various amino acid moieties which are not close to pH neutrality. Thus amino acids are much more capable as chemical catalyst for this reason. Without the participation of amino acids, ribozymes must attain complex folds, often with some dynamic character (pseudoknots) to be effective catalyst allowing them to cleave and ligate RNA. Given this chemical advantage, we might expect that RNA evolution was greatly facilitated (but not coded) by peptides that contribute catalytically, as stabilizers or selectors for specific RNA sequences [157]. In addition, such a modified RNA would likely also provide a chemical marker that could distinguish this RNA population. Indeed this molecular identity idea is developed below as a way to better understand the origin of tRNA and its role in initiating replication of so many RNA viruses, as well as how this chemical marker could promote the natural genetic code.

## 16. Emergence of RNA group identities

A good starting point for the accumulation of complexity seems to be hairpin ribozymes whose activity can be controlled by external effectors [158]. Structural variation in these ribozymes allows progeny RNA to have different functions from their parental RNAs. The objective is to replicate RNA with RNA which hairpin ribozymes can perform via a sequence of ligation reactions that produce a longer ribozyme [159]. Along these lines, two short hairpin RNAs can catalyze their own ligation to form larger RNA constructs [160]. Thus we see interactions that promote more complex progeny. However, for a fully active ribozyme, complex RNA folding is needed. And such folding is cooperative [161]. Folded ribozymes can also interact with other small molecules promoting their function as riboswitches [162],[163]. This includes amino acids which could promote either catalytic control or group identity marking [164]. And the ribozyme folds can also be dynamic and context sensitive as seen in pseudoknots [165]. But ribozymes can also be invasive, including self invasive [166]. Thus stem-loop RNA have many behaviors that would allow them to function as a mixture of agents involved in their own identification and synthesis [167],[168].

Of particular interest is their ability to self ligate as this could promote the emergence of social RNA networks with group identity [169],[170]. We can also think of tRNA as stem-loop RNA with various functions and histories. Indeed, it appears that tRNAs evolved from two separate hairpins [171], in which each of the stem loops interacts with a different ribosomal RNA subunit. This is a very interesting observation from an social RNA network perspective. The invasive nature of intron ribozymes (endonuclease) also applies to tRNA from archaea, but here four distinct specificities are known [172]–[174]. This very much resembles an identity system in which introns are marking central cellular (self) agents (such as tRNAs) for group identity but should destroy similar tRNAs lacking the intron marking. It is thus also interesting that tRNA with various linked amino acids themselves have been proposed to have originated before the translation system as genomic 3′ tags needed for RNA ribozyme replication [175]–[178]. This early function can also be explained as having served as a tag for group identity and could better explain the polyphyletic nature of the origin of tRNA [179].

## 17. Network membership–basal need to belong

The perspective social interacting networks of quasi-species consortia allows us to consider the role of stem-loop RNAs in the origin of the RNA world in which the action of individual agents can cooperate and be combined into a more capable collective action of a population. Thus the origin of spontaneous cooperating networks of stem-loop RNA replicators can be understood from this perspective [41]. However, we will use the term network to include some distinct features specifically network membership. To designate this situation we apply the term social network to distinguish networks that have no social membership criteria.

Basically, for a network to be coherent and able to act collectively, it must limit membership to promote coordination by communication [43]. Otherwise it is simply a abiotic collection of uncoordinated entities and there will be no selection for maintaining the network existence. If we are examining a network composed of stem-loop RNAs, it will be necessary for the individual RNAs to have some behaviors that maintains membership such as replication and identification of self and non-self members. As mentioned by Nelson and Breaker this requires signal mediated interaction. If only one type of RNA is supported (e.g. high fidelity replication), there can be no complementation and complex function (i.e. ribozyme) for the collective. A diversity of behavior and type will be essential. Recall however, that these RNAs act as agents in which various (multiple) behaviors will be possible even for the same sequence. This means there is diversity of interaction as well as diversity of type. Thus overall interaction of an RNA agent with the collective must promote coherence and continued existence. What then are the features that promote continued existence (selection) for a social network?

This does not require that only positive (e.g. replication) interactions be supported. Negative interactions, including interference will also be needed. For example, highly efficient run-away replicons would overtake a QS collective and yield only one RNA type. Thus the QS would lose complementing functionality and would also consume all substrates if they were not regulated. This situation presents a problem in those habitats with limited substrates, likely a very common state. Therefore, some level of self regulation (negation) in the collective would promote the survival of the collective, especially if these RNAs could interact with the substrate in a regulatory (e.g., riboswitch) manner. That efficient replicators become susceptible to parasitic replicators would provide an inherently spontaneous process of self regulation. Yet the collective will still need to promote replication when it is favored. Accordingly, it becomes important for members of the collective to be subjected to both positive and negative self regulation via RNA-RNA interactions. However, here too there must be some limits to self regulation as the collective cannot tolerate overly active self regulating members that will extinguish the collective.

Thus we see that being a successful member of a collective has many (and multiple) behaviors associated with it. On top of that, as a QS-C replicates, these features will drift with time in a dynamic manner. In this context we can see that a random RNA stem-loop or a stem-loop RNA from a different QS collective would likely not be coherent with the other members of a particular QS. A social QS network is generally rather specific for its members. Group selection has already started. Indeed, many experiments with RNA viruses infecting humans and animals have shown a particular QS will exclude other QS of the same virus which results into immune function. Additionally such society membership is also time dependent in that the serial passage of the same viral QS will usually result in subsequent QS that preclude prior individual members of the QS. This behavior has often been called a ‘Red Queen’ behavior, but such a classical neo-Darwinian view does not incorporate or acknowledge the issue of group membership [180]. The membership view, on the other hand, allows us to understand the maintenance of minority types in the collective since these members can provide a needed but complementing catalytic control. Thus a social QS consortia is a network that will naturally promote the emergence of membership. And as noted, defective interfering agents can also contribute to membership control.

## 18. To be or not to be part of group identity

As previously proposed group membership can also be promoted by the combined action of toxic agents linked to antitoxic agents [72]. A common version of a toxic agent is an endonuclease that will cleave sequences that are identified as foreign. The antitoxin in this case prevents the action of the endonuclease e.g., via a bound protein or methylated base, dsRNA with another molecule, altered RNA fold etc. In this light, the endonuclease and ligation activities of stem-loop ribozymes are particularly interesting [181]. A stem-loop ligase could identify non-member stem-loop RNAs and destroy them by ligation. Recall, however, that serial ligation can also be used to copy a stem-loop RNA. But such a situation has several very interesting implications. One of the problems with a group identity of stem-loop RNAs is that to attain their combined function, they need precise physical molecular placement relative to one another. This would normally require a high concentration dependence to counteract diffusion. By ligation, however, we could build a group identity of stem loop RNAs that have covalently placed the various stem-loops in the correct functional (or dynamic/regulatory) context and have lost their concentration dependence. It seems likely that such a process would involve invasive self colonizing stem-loop RNAs that results in one molecular entity with a common identity function. This would generate one entity that evolved from the ligation of a mixed set of stem-loop agents that now have a highly enhanced (collective) functional capacity. This collective would also have a highly enhanced capacity for persistence as it need not continually replicate individual stem-loop RNA agents to maintain its membership. The collective, however, would still need to oppose non-members or other parasite participation. Additionally, a collective might attain a conditional (regulated) replication capacity if it incorporates stem-loop RNA riboswitches. It is by such a process that we can now consider the origin of the ribosome [182],[183].

Membership is thus crucial for living networks to emerge. In examining the literature relevant to QS, the RNA world and RNA network formation, we can indeed find some experimental evidence that supports QS and the spontaneous emergence of RNA networks [41],[160]. But almost completely lacking from such experiments is any evaluation of the membership issue. For example, quasispecies-like behavior has been observed with in vitro RNA replicator studies [184]. Nonenzymatic template (peptide) directed autocatalytic systems can show network behavior [185],[186]. And communities of RNA ribozyme replicator sets can also show lateral evolution [187]. Also rule based computing simulation have been applied to similar systems in an effort to understand the emergence of parasites and antiparasites [10],[188]. Along these lines, the hypercycle kinetic model was proposed to be a system of cross catalyzing RNA replicators which depend on cooperation for growth [189]. But hypercycles as proposed are not able to tolerate networks of non-related parasites, let along depend on them for development.

Yet the biggest problem of all such studies is that there are few assumptions regarding the basic importance of social network membership [190]. Without a social network membership concept and its attending strategies, authentic collective action does not emerge. The dynamic nature of network membership and collective action poses many unsolved problems for existing theory. For example, how is the multi-potential of an individual RNA to be evaluated within the QS-C if we cannot specify all the other interactions and how they change with time? We cannot apply our current ideas of fitness to this individual RNA as the historical and population context is key [191],[192]. Increasing research on history and context of such QS-C are detecting the pathways how RNA stem loops with different ancestral history contribute to newly arising consortia [193],[194].

Network membership needs to be prominently considered if we are to understand the origin of the ribosome and the genetic code. The emergence of the genetic code is not solely a molecular biological invention but the result of social interacting RNA consortia which needed many code using agents [195],[196]. For in contrast to neo-Darwinian evolution, network members will generally have distinct ancestral histories. These members will mostly originate from separate parasitic lineages that were able to penetrate defenses and join the network, sometimes in mixtures. They don't need to descend from one individual or even be from the same type of agents such as virus, transposon, intron, inteines and others [197]–[199]. From this perspective we can understand why the two halves of tRNA have distinct evolutionary histories, yet tRNA is a core agent for evolution of life [200],[201]. Thus neither the amino acid based (peptide) ancestors or the RNA based ancestors need a common origin to participate in a symbiogenic network. [202],[203].

## 19. Conclusions

The perspective on social networking of quasi-species consortia (qs-c) provides us with the opportunity to coherently explain how RNA stem loop groups mediate evolutionary novelty and genetic variations. The presented contribution basically traces social stem-loop RNAs from providing replicators, marking group identity, to the invention of ribosomes and translation. The half a century outlasting quasi species theory with its individual fittest type selection narrative is now revisited into social networking of quasi species consortia that provide us with a model how communicative interacting RNA groups generate genetic novelty, transfer even complex genetic content to cellular host organisms via infectious genetic parasites and evolutionarily change their genetic identity by persistence. The error-replication narrative of the last century can be replaced now by current knowledge about creative productivity of quasi-species consortia. The crucial roles of quasi species consortia membership which includes also minority types elucidates how such RNA populations may compete and cooperate in parallel in an indefinite and unpredictible way. As main drivers of genetic novelty they represent virolution being the essential pathway of the evolution of the tree of life.
